# Operationalizing Clinical Speech Analytics: Moving From Features to Measures for Real-World Clinical Impact

**DOI:** 10.1044/2024_JSLHR-24-00039

**Published:** 2024-06-05

**Authors:** Julie Liss, Visar Berisha

**Affiliations:** a Arizona State University, Tempe

## Abstract

**Objective:**

This research note advocates for a methodological shift in clinical speech analytics, emphasizing the transition from high-dimensional *speech feature* representations to clinically validated *speech measures* designed to operationalize clinically relevant constructs of interest. The aim is to enhance model generalizability and clinical applicability in real-world settings.

**Method:**

We outline the challenges of using conventional supervised machine learning models in clinical speech analytics, particularly their limited generalizability and interpretability. We propose a new framework focusing on speech measures that are closely tied to specific speech constructs and have undergone rigorous validation. This research note discusses a case study involving the development of a measure for articulatory precision in amyotrophic lateral sclerosis (ALS), detailing the process from ideation through Food and Drug Administration (FDA) breakthrough status designation.

**Results:**

The case study demonstrates how the operationalization of the articulatory precision construct into a quantifiable measure yields robust, clinically meaningful results. The measure's validation followed the V3 framework (verification, analytical validation, and clinical validation), showing high correlation with clinical status and speech intelligibility. The practical application of these measures is exemplified in a clinical trial and designation by the FDA as a breakthrough status device, underscoring their real-world impact.

**Conclusions:**

Transitioning from speech features to speech measures offers a more targeted approach for developing speech analytics tools in clinical settings. This shift ensures that models are not only technically sound but also clinically relevant and interpretable, thereby bridging the gap between laboratory research and practical health care applications. We encourage further exploration and adoption of this approach for developing interpretable speech representations tailored to specific clinical needs.

In 2013, we developed a supervised learning model trained to predict perceptual labels (articulatory precision and hypernasality) from high-dimensional feature representations. The speech corpus contained thousands of sentences and phrases produced by roughly 100 speakers with various subtypes and severities of dysarthria. In this context, the “ground truth” of the model was the set of perceptual rating labels (provided by speech-language pathologists [SLPs]) for each of these sentences and phrases, against which our model's predictions were gauged, serving as a benchmark for its accuracy. The “high-dimensional” nature of our feature representations refers to the vast array of attributes derived from the speech samples in the temporal and spectral domains, which our model utilized to make its predictions. Furthermore, our model operated as a “black box,” in that, while it could predict labels with a certain degree of accuracy, the internal mechanics—how it arrived at these predictions—remained obscured.

Following model development, our clinical colleagues collected speech from their clients with dysarthria at the Arizona State University Speech Clinic and we tested our model's performance. Disappointingly, our model did not generalize at all on these new speakers. In fact, it failed miserably in predicting the perceptual rating labels. While the model demonstrated a correlation of .75 between the predicted labels and the ground truth perceptual labels on our test sample, that reduced to ~.4 when evaluated on the prospectively collected data. This variability made the model unusable. We posit that our story is not unique. Subsequently, there have been several studies that demonstrate that the analytical flexibility provided by machine learning runs the risk of generating overoptimistic estimates of model performance during development and failure postdeployment (Berisha et al., 2021, 2022; Coretta et al., 2023; Yawer et al., 2023).

Reflecting on this setback, we derived important lessons that directly inform our subsequent argument regarding the inadequacy of current input representations in clinical speech models. The inherent opacity of our “black box” machine learning models and the lack of a clear link between model features and underlying clinical constructs of interest (in this case, the constructs of articulatory precision and hypernasality) prevented any insight into the failure mechanisms, illustrating a fundamental flaw when applied in clinical settings where understanding and trust are paramount. This misalignment is a critical issue that we address by proposing a shift toward more clinically relevant input representations. Furthermore, the comparison of what constitutes a “large” data set in clinical research versus consumer technology applications revealed a disparity, highlighting the challenge of achieving generalizability with limited clinical data. This challenge further justifies the need for a new approach to input representation in clinical speech models, emphasizing the transition toward individually validated and interpretable speech measures that reduce analytic flexibility in the models by constraining their solution space.

It is common in many clinical speech publications for researchers to use high-dimensional speech feature representations, such as mel-frequency cepstral coefficients, that are often repurposed from other applications like automatic speech recognition or audio engineering (Stegmann, Hahn, Liss, Shefner, Rutkove, Kawabata, et al., 2020). Clinical grade models for high-stakes clinical use cases require stringent validity and reliability criteria that are often unattainable with general-purpose, high-dimensional feature sets (Fleiss, 1999; Stegmann, Hahn, Liss, Shefner, Rutkove, Kawabata, et al., 2020). In this research note, we argue that a new input representation is required for clinical speech analytics to generalize, one that shifts from uninterpretable speech *features* to individually validated speech *measures* designed to operationalize a speech construct of interest as an objective speech measure. This is not a new concept as measurement is a hallmark of scientific inquiry. However, in the following sections, we submit that applying measurement theory in the context of machine learning model development for clinical speech applications can improve generalizability, transparency, and translation, illustrated by a case study detailing the development, validation, and clinical application of a measure for articulatory precision for amyotrophic lateral sclerosis (ALS).

## Moving From Speech Features to Speech Measures

In clinical speech analytics, the relationship between speech constructs and clinical conditions is a primary focus:

- *Prosody* is often affected in autism spectrum disorders, schizophrenia, and nonfluent aphasia.
- Children with cleft palate commonly exhibit *hypernasal* speech.
- Motor conditions like Parkinson's disease and ALS are frequently associated with reductions in *articulatory precision*.
- Parkinson's disease often results in a breathy voice, thereby impacting *vocal quality*.

**Figure 1.**
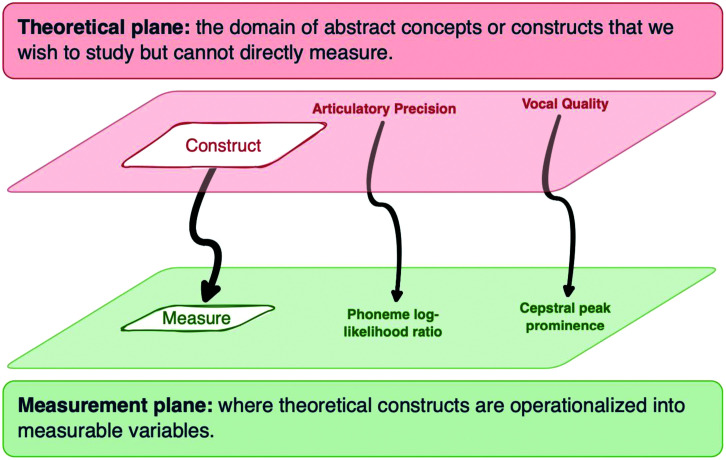
Diagram showing the transition from theoretical constructs (e.g., articulatory precision, vocal quality) to practical measures (e.g., phoneme log-likelihood, cepstral peak prominence) in clinical speech analytics. Speech measures can be empirically validated relative to the constructs they are designed to assess.

These statements articulate relationships between clinical conditions and their effects on various speech constructs. However, these constructs often lack direct, measurable counterparts and remain within the theoretical plane in Figure 1—the domain of abstract concepts not directly observable or quantifiable. For example, one might describe someone's articulatory precision as being crisp and clear, or slurred and mumbled. The relationship between the speech construct and speech acoustics is complex and not straightforwardly quantifiable. Therefore, especially for high-stakes clinical applications, it is crucial to operationalize the abstract construct as an objective measure whose validity and reliability can be scrutinized. In the figure, we show that one way to operationalize the speech constructs of articulatory precision and vocal quality is as measures of mean log-likelihood ratio and cepstral peak prominence, respectively, drawing from the rich clinical speech science literature on speech and voice acoustics (Riesgo & Nöth, 2020; Stegmann et al., 2023). The operationalization of these constructs is imperative to bridge the gap to the measurement plane, where theoretical constructs are translated into observable, quantifiable variables (Allen & Yen, 2001). This transition is crucial for empirical testing, validation, and application in clinical settings.

In traditional clinical speech science, the transition from the theoretical plane to the measurement plane is careful and deliberate. For instance, in motor speech disorders, theoretical constructs are operationalized through perceptual ratings by trained experts, as validated and assessed for reliability across contexts with recognized limitations (Bunton et al., 2007). Such measures are fundamental in speech pathology, providing frameworks for diagnosis and categorization of speech disorders (Duffy, 2020).

On the other hand, in clinical speech analytics, there is a notable gap in rigor when transitioning from the theoretical to the measurement plane and using representations with established construct validity. Established theoretical constructs such as prosody, hypernasality, and articulatory precision are well documented but are often complex and not straightforwardly quantifiable. Researchers working on machine learning model development rely on high-dimensional feature representations, either explicitly extracted or implicitly learned through deep learning, that capture a wide array of speech characteristics that may be related to the specific theoretical construct, but are not directly developed to measure it. This approach presents several issues. Utilizing features without construct validity compromises interpretability, a critical aspect of model development in clinical applications. Furthermore, high-dimensional, nonspecific features lack stability (Stegmann, Hahn, Liss, Shefner, Rutkove, Kawabata, et al., 2020), yielding unreliable models—a significant impediment to practical deployment (Berisha et al., 2021, 2022).

**Figure 2.**
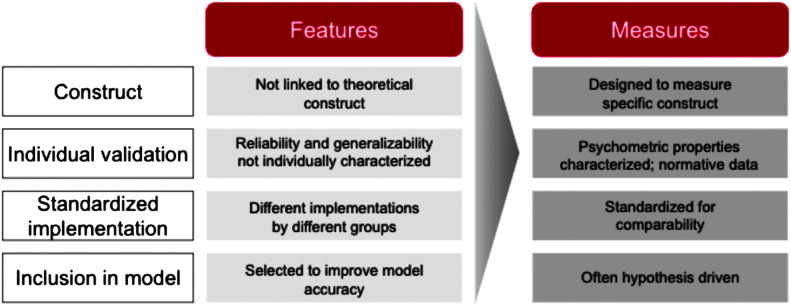
Comparative overview between speech features and speech measures: Speech features often lack a direct link to theoretical constructs and vary in implementation, while speech measures are tailored to capture specific constructs with established psychometric properties, enabling standardized and hypothesis-driven inclusion in clinical models. The shift to speech measures is less relevant for nonclinical models with large amounts of speech data is available. In these cases, data-driven approaches with uninterpretable feature inputs have demonstrated success in applications like automatic speech recognition.

In Figure 2, we draw a distinction between speech features and speech measures and argue for an approach grounded in measurement theory to attain clinical grade models in the absence of large-scale labeled clinical data (Allen & Yen, 2001). Unlike features, measures are designed to quantify specific constructs, enhancing interpretability and reflecting a deeper understanding of the phenomena. Measures undergo psychometric validation, ensuring reliability and validity across different populations and contexts. Standardization of measures promotes consistency and study replication, in contrast to the disparate implementations of features (which may have different implementations by different labs). Measures integrated into models are hypothesis-driven, derived from theoretical frameworks or empirical evidence, which bolsters model interpretability and clinical applicability. By aligning model development with well-defined and validated measures, researchers can significantly reduce sample complexity, as models become more focused and efficient in learning from data that directly relates to the underlying clinical constructs. This targeted approach allows for the construction of models that require fewer examples to achieve high levels of accuracy and generalizability, thereby making the most of limited clinical data sets.

Without a tangible link between the measurement plane and theoretical constructs, model development and troubleshooting become difficult, detaching machine learning models from the wealth of existing clinical research. During model development, this approach facilitates the integration of existing know-how from clinical speech science within machine learning models, enhancing their applicability and effectiveness in real-world settings. This integration helps reduce analytic flexibility in models (creating improved conditions for generalization with smaller sample sizes) and results in models grounded in clinical reality. For model validation, bridging this gap between theoretical constructs and measurable outcomes enables the creation of more accurate, reliable, and clinically relevant machine learning tools.

This discussion highlights an opportunity for the clinical speech analytics community. There has been a recent explosion in the tools and foundational models available for speech analysis. Directing these tools toward the development of validated, standardized, and ever-improving measurement models for speech analytics can move the field forward. Concentrating on speech measures that are pertinent to clinical contexts can significantly advance the development of novel interpretable representations that can be extended across clinical use cases. Measures that are dimensionally concise, clinically pertinent, and validated on an individual basis, when rooted in a clinical framework, are more likely to produce stable and generalizable clinical models built on top of these measures.

## Case Study: Articulatory Precision in ALS

We were involved in several large-scale, longitudinal studies of individuals with ALS, including noninterventional natural history studies and clinical trials to test the impact of pharmaceutical agents on disease/symptom progression. In each of these studies, participants provided speech samples frequently (daily to several times a month) using a mobile application downloaded onto their smartphone or tablet. They were prompted to read aloud 10 sentences, sustain an “ah” phonation for as long as possible, and repeat the word “buttercup” as quickly and crisply as possible for several seconds. The recorded speech samples were uploaded to secure cloud storage and automatically analyzed to extract validated speech measures that were used to identify deterioration or improvement in speech and to develop clinical models. The purpose for presenting this case study is not to review the details of the methods and findings of these studies. Interested readers are encouraged to access the original publications of this work (Rutkove et al., 2019, 2020; Steggmann et al., 2023; Stegmann, Hahn, Liss, Shefner, Rutkove, Kawabata, et al., 2020; Stegmann, Hahn, Liss, Shefner, Rutkove, Shelton, et al., 2020). Instead, the purpose of this presentation is to demonstrate the successful development of one speech measure using artificial intelligence, from ideation through validation, and to a Food and Drug Administration (FDA) breakthrough status designation.

### Construct of Interest

In these longitudinal studies, we extracted a suite of measures for validation, including those related to respiratory support, phonation, articulation, resonance, and prosody. For a concise illustration, here we focus on the single construct of *articulatory precision*, keeping in mind that the other validated measures in the suite each underwent the same protocol. Recall that the construct of articulatory precision refers to a range of subjective impressions one experiences when they hear speech, from crisp and clear to slurred and mumbled. Physiologically and acoustically, articulatory precision is mediated by the integrity of the articulatory shapes and contacts (e.g., lips, tongue, soft palate) as they valve the voiced and unvoiced energy through the vocal tract. Speech with high articulatory precision contains vowels and consonants with maximum acoustic and perceptual distinctiveness. Low levels of articulatory precision challenge listeners to understand the words being said, and this directly results in poor speech intelligibility. Thus, improving articulatory precision to benefit speech intelligibility is standard practice in speech-language pathology (Duffy, 2020).

Unlike laymen, SLPs are highly adept at homing in on specific perceptual characteristics associated with articulatory precision, such as weak articulatory contacts (stops sound like fricatives), reduced vowel space (vowels lack perceptual distinctiveness and tend to sound schwa-like), and hypernasality effects on consonants (nonnasal stops sound like their nasalized cognates). SLPs often use perceptual ratings as operationalized measures to quantify the amount of aberrancy they hear. However, the link to objective acoustic measures of these constructs is less straightforward. It is highly challenging to measure acoustically, and it is even more challenging to measure it reliably, as is required for clinical grade speech analytics. This is because, as stated earlier, the construct is complex and not straightforwardly quantifiable. Therefore, our challenge was to move from the theoretical plane (articulatory precision as a construct) to the measurement plane (an objective measure of articulatory precision) by operationalizing this construct.

### Operationalizing the Construct

Articulatory precision can be operationalized through an algorithm that compares the observed acoustic output for phoneme production against a normative benchmark (Stegmann, Hahn, Liss, Shefner, Rutkove, Shelton, et al., 2020; Tu et al., 2018). In this case, the input to the algorithm is a set of audio recordings of read sentences remotely collected from a speaker with ALS, and the target transcript for each corresponding sentence. This measure uses an algorithm that evaluates how well the spectral representation of each observed phoneme in the speech recording corresponds to that of the expected phoneme, as represented in speech samples for a large normative population.

Targets for these acoustic benchmarks are determined using a trained acoustic model that captures phoneme representations within their phonetic context—a triphone model that accounts for adjacent phonemes. The acoustic model encodes the acoustic features within a neural network, trained on an extensive data set of American English speech (Panayotov et al., 2015). Upon analyzing a new speech sample, the network computes a likelihood ratio, comparing the actual phoneme production to the expected normative model. This ratio serves as the index of articulatory precision, with a score of 0 indicating precise articulation analogous to the normative model. Negative values, conversely, signify imprecise articulation and indicate a deviation from the distribution of acoustic patterns produced by a large sample of general population speakers. An aggregate score of articulatory precision is computed by averaging the precision scores of all phonemes and segments produced by an individual, thus yielding a singular, comprehensive measure of articulatory accuracy for each participant. By this method, we create an objective articulatory precision speech measure that can be evaluated for its reliability and validity.

### Validating the Measure

Best practices for the validation of digital measures have been proposed by the Digital Medicine Society (Goldsack et al., 2020). Specifically, completion of three evaluation modules (verification, analytical validation, clinical validation), termed the “V3 framework,” must be completed to demonstrate the validity of digital measures.

The first module is *verification* of the high fidelity of the sensors and hardware being used. Prior to measure validation, we conducted bench tests to verify that the hardware used to acquire the data provided speech samples of sufficient quality for accurate measurement of articulatory precision. This also provides reporting parameters for publications to enhance reproducibility by other research labs.

The second module is *analytical validation*, or does the analysis measure the construct it portends to measure. For analytical validation of our objective articulatory precision measure, perceptual ratings of articulatory precision on a 0 (*normal*) to 4 (*severely impaired*) scale were obtained from multiple SLPs. Weighted averages of these perceptual ratings served as the ground truth against which the articulatory precision measures were compared. For clinical grade analytics, the correlation between the digital measure and ground truth should be high, and it was in this case (*r* = .9; Stegmann et al., 2023). Furthermore, the digital measure should be stable on consecutive days if there has been no change in clinical status. High repeatability, or test–retest reliability (intraclass correlation coefficient = .97; Stegmann et al., 2023), was found for the measure of articulatory precision among the speech samples from participants with ALS.

The third module is *clinical validation*, demonstrating that the proposed measure tracks with quantifiable clinical standard ground truth measures. The ALS Functional Rating Scale–Revised (ALSFRS-R) is a clinically validated 0- to 4-point patient reported outcome scale that is a widely accepted end point for clinical trials in ALS. Articulatory precision correlated well with the ALSFRS-R speech item score (*r* = .82; Stegmann et al., 2023), and it declined over time with disease progression without intervention; the rate of decline correlated moderately with the rate of decline of the ALSFRS-R Speech (*r* = .37) and Bulbar (*r* = .41) subscales, which includes a speech item (Stegmann et al., 2023). Thus, based on the V3 framework, the digital measure of articulatory precision demonstrates both analytical and clinical validity and has high test–retest reliability.

### Establishing Clinical Meaningfulness

In addition to demonstrating validity and reliability of the digital measure, it must also be clear that changes in the measure are important and meaningful to patients. The literature and patient-reporting patterns reveal that difficulty speaking and being understood is a serious threat to quality of life for people with ALS (Börjesson et al., 2021). Reductions in articulatory precision are directly linked to reductions in speech intelligibility and increases in listener effort (Stipancic et al., 2018). This slippery slope leads to increasing social isolation and the eventual need for augmentative or assistive communication devices (AAC). The measure of articulatory precision has been shown to be predictive of the rate of speech decline, which can be useful in planning the timeline for introducing AAC (Stegmann et al., 2023). Furthermore, the measure has been shown to be predictive of intelligibility and communicative participation (Borrie et al., 2022).

### Real-World Clinical Use

The rigorous process by which this measure of articulatory precision was developed (along with a host of other validated measures developed as part of the longitudinal ALS studies) proved critical in identifying a positive treatment effect in a clinical trial of the drug, pridopidine, in ALS. In the Healey ALS Platform Trial at Massachusetts General Hospital, a prespecified analysis of a set of speech measures showed improvements in speech measures in participants taking pridopidine compared to those taking placebo (Massachusetts General Hospital, 2023). This finding was especially important because the primary end points (ALSFRS-R and survival) were not significant, nor were the secondary end points (muscle strength and respiratory function). The robust positive speech findings contributed to the decision to pursue a Phase 3 clinical trial of pridopidine in ALS (Prilenia Therapeutics, 2024).

### FDA Breakthrough Status Designation

The reliability and validation data coupled with evidence identifying the clinical meaningfulness of the measure were combined into an application for the FDA's Breakthrough Designation Program. The FDA Breakthrough Designation Program is designed to expedite the development and review of innovative medical products, such as medical devices and drugs, that have the potential to provide significant benefits over existing treatment for serious or life-threatening conditions. The rigor with which the speech measures were developed and vetted for analytical validity, clinical validity, test–retest reliability, and clinical meaningfulness provided a rich body of scientific evidence for the FDA to evaluate. They granted this technology Breakthrough Designation in March 2023.

## Discussion and Conclusions

In this research note, we highlight the challenge of building robust clinical grade speech models in the current absence of adequately large, labeled clinical data sets. We then call for a shift in clinical speech analytics away from conventional machine learning models using high-dimensional speech *features* to those using individually validated speech *measures* designed to evaluate clinically relevant constructs of interest. Key to this approach is the operationalization of speech constructs, moving them from the theoretical plane to the measurement plane. Such measures are then amenable to evaluation of validity, reliability, and clinical meaningfulness. In our case study, we demonstrate how large speech data sets generated by the general population can be leveraged for operationalizing speech measures in ALS dysarthria; in this case, we operationalized articulatory precision as a phoneme log-likelihood ratio measure. Finally, the research note describes a case study of the development and deployment of an articulatory precision measure in the context of ALS as a demonstration of how this approach can bridge the gap from research in the laboratory to real-world impact. As the case study demonstrates, the adherence to rigorous validation standards, as outlined in the V3 Framework, is a critical component of this approach of creating clinical grade speech tools. Concentrating on speech measures that are clinically relevant will significantly advance the development of interpretable models across clinical use cases.

Future research should explore the extent to which the methodology used in this study can be generalized across a broader spectrum of clinical populations. While ALS is a natural initial use case for demonstrating the approach (and the clinical utility of speech analytics), other clinical conditions may be more challenging. For example, the variability in speech characteristics among individuals with neurodevelopmental conditions, such as autism spectrum disorder, presents a unique challenge for the development of clinical speech measures (Rybner et al., 2022). Nevertheless, working toward interpretable, individually validated measures will enable the creation of more robust, generalized models with potential for real-world impact.

## Notes

**Publisher Note:** This article is part of the Forum: Research Symposium on Artificial Intelligence.
